# Lgr5 Methylation in Cancer Stem Cell Differentiation and Prognosis-Prediction in Colorectal Cancer

**DOI:** 10.1371/journal.pone.0143513

**Published:** 2015-11-24

**Authors:** Shasha Su, Feng Hong, Yanling Liang, Jieqiong Zhou, Yan Liang, Kequan Chen, Xinying Wang, Zhongqiu Wang, Zhiqing Wang, Cassie Chang, Weihua Han, Wei Gong, Haitao Qin, Bo Jiang, Huabao Xiong, Liang Peng

**Affiliations:** 1 Guangdong Provincial Key Laboratory of Gastroenterology, Department of Gastroenterology, Nanfang Hospital, Southern Medical University, Guangzhou, 510515, China; 2 Institute of liver diseases, Affiliated Hospital of Jining Medical University, Shandong, 273100, China; 3 Immunology Institute, Department of Medicine, Icahn School of Medicine at Mount Sinai, 1 Gustave L. Levy Place, New York, New York 10029, United States of America; 4 Second affiliated hospital of XingTai medical college, Hebei, 054002, China; University of Navarra, SPAIN

## Abstract

**Objective:**

Leucine-rich-repeat-containing G-protein-coupled receptor 5 (lgr5) is a candidate marker for colorectal cancer stem cells (CSC). In the current study, we investigated the methylation status within thelgr5 promoter and evaluated its relationship with CSC differentiation, prognosis for colorectal cancer, and its clinicopathological features.

**Methods:**

The methylation status within Lgr5 promoter was detected with a methylation-specific PCR in six colorectal cancer cell lines as well as 169 primary colorectal tumor tissues. Differentiation of CSC was examined with immunofluorescence and immunocytochemistry. Down-regulation of lgr5 was achieved with gene-specific siRNA. The associations between lgr5 methylation and the clinicopathological features as well as survival of patients were analyzed with statistical methods.

**Results:**

The lgr5 promoter was methylated to different degrees for the six colorectal cell lines examined, with complete methylation observed in HCT116 cells in which the lgr5 expression was partially recovered following DAC treatment. The stem-cell sphere formation from HCT116 cells was accompanied by increasing methylation within the lgr5 promoter and decreasing expression of lgr5. Knocking down lgr5 by siRNA also led to stem-cell spheres formation. Among primary colorectal tumors, 40% (67/169) were positive for lgr5 methylation, while none of the normal colon tissues were positive for lgr5 methylation. Furthermore, lgr5 methylation significantly associated with higher tumor grade, and negative distant metastasis (p < 0.05), as well as better prognosis (p = 0.001) in patients with colorectal cancer.

**Conclusions:**

Our data suggests that lgr5 methylation, through the regulation of lgr5 expression and colorectal CSC differentiation, may constitute a novel prognostic marker for colorectal cancer patients.

## Introduction

Accumulative evidence supports the hypothesis that a small number of undifferentiated stem or stem-like cells, so-called cancer stem cells (CSC), are responsible for tumor initiation, development, maintenance, dissemination, regeneration and therapeutic resistance. Later on, the presence of CSC was confirmed in a variety of solid cancers, such as breast cancer[1, 2], glioblastomas[3], hepatocellular carcinomas[4], and so on, which was achieved with the help of, and in turn, promoted the identification of many tumor-specific cell surface antigens, also known as CSC markers. For colorectal cancer, several CSC markers have been identified, including CD133[5, 6], Epcam/CD44/CD166[7], CD24/CD29[8] and lgr5[9, 10]. However, little is known of the biological significance of these markers, which may have strong implications in the multilineage differentiation capacity of CSC[8].

Among the colorectal CSC markers, lgr5, also known as GPR49, is an orphan G-protein coupled receptor (GPCR) that belongs to the leucine-rich repeat-containing GPCR[11]. Recently, it was reported that lgr5 is positive in stem cells of the small intestine, colon and hair follicle[9, 12, 13], suggesting potential significance of lgr5 in stem cell biology. Consistently, the lgr5-expressing stem cells were capable of building organoid structures in vitro, which was experimentally demonstrated in the intestine[14–16], stomach[17] and liver[18]. Moreover, the recipient mice with superficially damaged colons were transplanted with organoids derived from a single Lgr5^+^ colon stem cell after extensive in vitro expansion, consequently formed self-renewing crypts that were functionally and histologically normal[15]. On the contrary, the lgr5-null mice exhibited neonatal lethality due to ankyloglossia and gastrointestinal distension[19]. The up-regulation of lgr5 has been reported in many human solid tumors, such as hepatocellular carcinomas[20, 21], colon[22], ovarian tumors[22], basal cell carcinoma[23] and gastric cancer[24]. Functionally, enhanced lgr5 expression promoted cancer cell proliferation and tumorigenicity[23], while the silencing of lgr5 reduced proliferation, migration and colony formation, tumorigenicity[25] and induced cellular apoptosis[22]. Lgr5 is a downstream target for the Wnt-β-catenin signaling pathway[26]. In intact animals, lgr5 was a part of a negative feedback loop fine-tuning Wnt signaling during intestinal morphogenesis. Lgr5 deficiency induced premature differentiation of Paneth cells, which is concomitant with the up-regulation of Wnt activity, implying lgr5 is a negative regulator of Wnt signaling in progenitor cells of the developing intestine[27]. In addition, lgr5 homologues are facultative Wnt receptor components that mediate Wnt signal enhancement by soluble R-spondin proteins[28].

Increasing evidence points to the importance of lgr5, at different expressional levels, in various disease paradigms and developmental stages. However, the underlying mechanisms for expressional regulation on lgr5 still remain elusive.

DNA methylation has been demonstrated as an important epigenetic mechanism for inactivating genes during tumourigenesis[29, 30]. Recently, there are ever-increasing researches that reveal that the methylation of some genes regulate development, progression, and recurrence of tumors[31–33], of which include colorectal cancer[34]. In the present study, we examined the methylation status of CpG islands within the immediate promoter region of lgr5 gene in colorectal cancer cell lines and tumor tissues, and analyzed its association with CSC differentiation, clinicopathological features, as well as the prognosis of patients with colorectal cancers.

## Materials and Methods

### Cell culture and treatment

Colorectal cancer cell lines (HCT116, SW480, HT29, LoVo, colo205, SW620, SW1116) were obtained from American Type Culture Collection (ATCC, Manassas, VA) and cultured in RPMI 1640 medium supplemented with 10% fetal bovine serum (Sijiqing, Beijing, China). For demethylation treatment, cultured cells were incubated with 3 μM 5-aza-2’-deoxycytidine (DAC, Sigma, St. Louis, MO) for 72h with medium changed every day. Mock drug treatments were performed in parallel with PBS (PH 7.4).

### Human tissue samples and patient information

This study was approved by the Ethics Committee of Southern Medical University (Guangzhou, China) and written consent was obtained from all participants. 169 patients (mean age 57±22.3 years) were diagnosed with sporadic colorectal cancer in Dept. of Pathology (Nanfang Hospital, Southern Medical University) between 1999 and 2001. All diagnoses were established based on histological examinations of standard HE-stained sections according to the WHO guidelines. Tumors were staged based on the Dukes staging system. These patients did not receive neoadjuvant radio-chemotherapy nor did they have any complicattions with other known malignancies at the time of diagnosis. Patients died within six months following surgical resection were excluded.

### Western blotting analysis

25 μg of the total protein was incubated with denaturing buffer (0.3 M Tris pH 6.8, 10% 2-mercaptoethanol, 40% glycerol, 20% SDS, 0.02% bromophenol blue) for 5 min at 95°C, loaded onto a 10% SDS-polyacrylamide gel for electrophoresis, transferred onto PVDF membranes, blocked in 5% non-fat milk/TBS-Tween for 1 h at room temperature and incubated overnight at 4°C in a primary antibody against lgr5 (1:500, Abcam, Cambridge, MA) or a mouse alpha-tubulin antibody (internal control, 1:1,000, Cell Signaling, Danvers, MA). Membranes were then incubated with horseradish peroxidase-conjugated secondary antibodies for 1 h at room temperature. Immunoreactivity was detected chemiluminescence (Pierce, Rockford, IL).

### RT-PCR

Total RNA was extracted with Trizol reagent (Invitrogen, Carlsbad, CA) and 2 μg total RNA was subjected to the first-strand cDNA synthesis (Fermentas, Ontario, Canada) according to the manufacturer`s instructions. Primer sequences for RT-PCR were listed in Table 1, with GAPDH used as an internal control.

**Table 1:** Primer sequences for RT–PCR, MSP and BSP analysis.

Gene	Primer sequence (5’-3’)	Amplification size (bp)
**RT-PCR**		
**Lgr5**	F: GAGGATCTGGTGAGCCTGAGAA R:CATAAGTGATGCTGGAGCTGGTAA	151
GADPH	F: CCAGCCGAGCCACATCGCTC R:ATGAGCCCCAGCCTTCTCCAT	300
**Lgr5 MSP**		
Methylation	F: CGGTAATCGGTATTTTTGTTTTC R:TAAACTTCTACAACTCAACGAACGT	108
Unmethylation	F: GGTGGTAATTGGTATTTTTGTTTTT R:AAACTTCTACAACTCAACAAACATC	109
**BSP**	F: GGGTGTTTGGGAAGTTAGGTT R:CAACTACAACAACACAAACAAAAAC	428

Note: F: forward primer; R: reverse primer; bps: base pairs.

### DNA extraction, methylation-specific PCR (MSP) and bisulfite sequencing PCR (BSP)

Genomic DNA was extracted from cell lines or primary tumors following a standard phenol-chloroform extraction method (Qiagen, Valencia, CA). Bisulfite modification of genomic DNA was carried out using the EZ DNA methylation kit (Zymo Research, Orange, CA). The methylation pattern in the CpG islands within lgr5 promoter was determined by MSP using bisulfite-treated DNA as the template and MSP primers listed in Table 1. MSP primers were designed following the principle as described previously[35] and tested for not amplifying non-bisulfited DNA. MSP was conducted at 95°C for 10 min, followed by 38 cycles at 94°C for 30s and 72°C for 30s, then by a 5-min extension at 72°C. The MSP products were then separated on 1.5% agarose gels stained with ethiium bromide and visualized under UV spectrophotometer. Water blanks were used as negative control.

For bisulfite sequencing, BSP primers (Table 1) were used to amplify a 428-bp fragment covering the promoter-exon 1 (-362 to +96) region of the lgr5 gene. The BSP primers were designed not to cover any CpG sites. The PCR products were then subcloned into pCR2.1-TOPO (Invitrogen) plasmids and the plasmid DNA from five bacterial clones were selected for DNA sequencing according to previous described[36].

### Stem cell sphere formation and differentiation

To examine stem cell sphere formation, 5 ×10^5^ HCT116 cells were cultured in 60-mm culture dish in suspension in serum-free DMEM/F12 (Gibco, Carlsbad, CA), supplemented with B27 (1:50, Invitrogen), 40 ng/mL EGF (Sigma) and 20 ng/ml FGF-2 (Chemicon, Billerica, MA),100U/mL penicillin G, 100ug/ml streptomycin (Gibco, Australia). CSC spheres started to form approximately two weeks later. To induce CSC differentiation, the CSC spheres were washed with PBS three times and then cultured in RPMI 1640 medium supplemented with 10% fetal bovine serum, 100U/mL penicillin G, 100ug/ml streptomycin. Nuclear acid and protein were extracted in from these cells on day 0, 1, 3 and 7, respectively as described[37–39]. All cells were cultured at 37 0C in a humidity atmosphere containing 5% CO2.

### Immunofluorescence (IF) and immunohistochemistry (IHC)

For IF, CSC spheres were fixed with 4% formaldehyde in PBS for 10 min at room temperature and then permeabilized with PBS containing 0.1% Triton X-100. After blocking with 1% bovine serum albumin for 20 min at room temperature, the CSC spheres were incubated with primary mouse monoclonal antibody CK20 (1:300, Santa Cruz, CA) at 4°C overnight and then with FITC-conjugated anti-rabbit lgG (Santa Cruz).

For IHC described as our previous methods [40], formalin-fixed paraffin-embedded tissue sections were dewaxed in xylene and rehydrated with distilled water.,and was performed heat mediated antigen retrieval with citrate buffer pH 6.0 and block with 3% BSA. The slides were subsequently incubated with the lgr5 antibody (1:300, #137484,Abcam) at 4°C overnight, with the signal amplified and developed using the ABC system and DAB substrate chromogen (Maixin Bio, China), respectively, following by haematoxylin counterstaining. Expression was considered to be “positive” when 10% or more cancer cells were stained.

### Transfection of small interfering RNA (siRNA)

The targeting sequence for lgr5-specific siRNA nucleotides was 5’-GATCTGTCTTACAACCTAT-3’ and the scrambled siRNA sequence 5’-UUCUCCGAACGUGUCACGU-3’ was used as the control. Both siRNA duplexes were synthesized by GenePharma (Shanghai, China). Transfection was performed using Lipofectamine transfection reagent (Invitrogen) according to the manufacturer’s instructions.

### Statistical analysis

To assess the independent correlation of lgr5 methylation with other variables, multivariate logistic regression analysis was performed. For survival analysis, Kaplan-Meier method was used to assess survival time distribution relative to lgr5 methylation. *p* value of less than 0.05 was considered statistically significant.

## Results

### Lgr5 was differentially methylated in colorectal cancer cell lines

**Fig 1:**
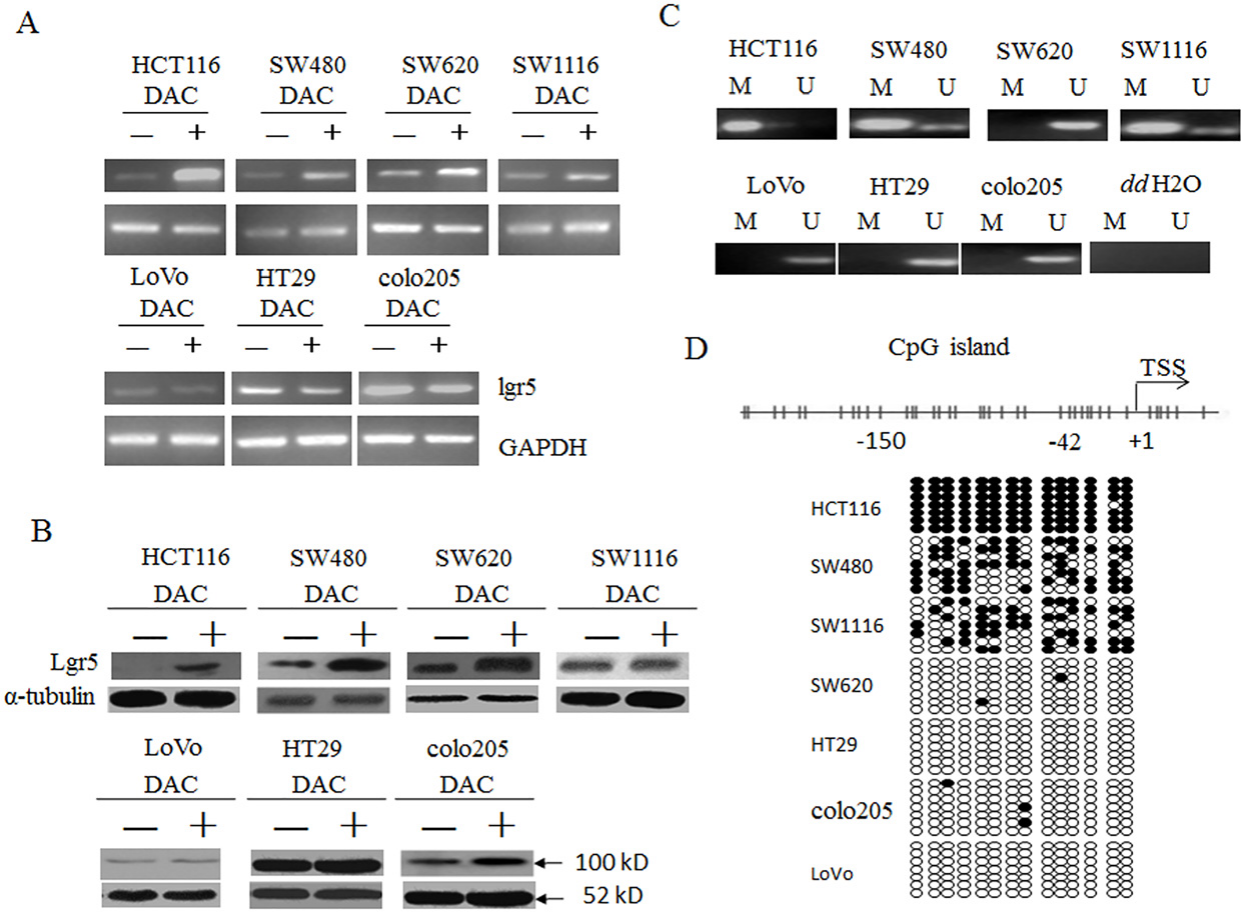
Lgr5 was differentially methylated in seven colorectal cancer cell lines. **A**. Total RNA was extracted from indicated cells without (-) or with (+) DAC treatment and RT-PCR was performed to examine lgr5 steady-state mRNA level, with GAPDH as an internal control. **B**. The protein level of lgr5 was measured in all seven colorectal cancer cells treated as in A with western blot analysis. α-tubulin was used as an internal control. **C**. DNA methylation status of a CpG island within the proximal promoter region of lgr5 gene was determined with MSP analysis. M, methylation signal; U, unmethylation signal. *dd*H2O, water containing no genomic DNA. **D**. Methylation density within the proximal promoter region of lgr5 gene in seven colorectal cancer cell lines was determined by BSP analysis. Upper panel, diagram of the distribution of CpG sites within the 5’ UTR of lgr5 gene. Each vertical bar represented a CpG site. Lower panel, BSP of 14 CpG sites within -150 to +42 region relative to the transcription star site (TSS) in seven colorectal cancer cell lines. Each row represents an individual cloned allele that was sequenced following bisulfite DNA modification. Circles represent CpG sites and their spacing accurately reflects the CpG density of the region. Black circle, methylated CpG site; white circle, unmethylated CpG site.

To test the potential of DNA methylation in regulating lgr5 expression, we first examined the expression of lgr5 in seven colorectal cancer cell lines and its alteration in response to 5-aza-2’-deoxycytidine (DAC), the classical methylation inhibitor. As shown in Fig 1A and 1B, for HCT116, SW480 and SW620 cells, lgr5 expression on both steady-state mRNA and protein level were up-regulated in response to DAC treatment, suggesting lgr5 expression might be inhibited in these cells via DNA methylation. In contrast, the expression in SW1116, LoVo, HT29 and colo205 cells was not dramatically altered following DAC treatment, implying an irrelevance to methylation-involved mechanisms. Consistent with these observations, we detected complete methylation of the promoter region CpG islands in HCT116 cells, partial methylation in SW480 and SW620 cells and no methylation in HT29, colo205, SW1116 or LoVo cells (Fig 1C) by MSP analysis. The accuracy of MSP assay was further confirmed with bisulfite sequencing (Fig 1D). Therefore, the expression of lgr5 in these cancer cell lines well correlated with the methylation status of the promoter CpG islands, supporting DNA methylation as a mechanism for regulating lgr5 level.

### Lgr5 methylation correlated with CSC- differentiation in vitro

**Fig 2:**
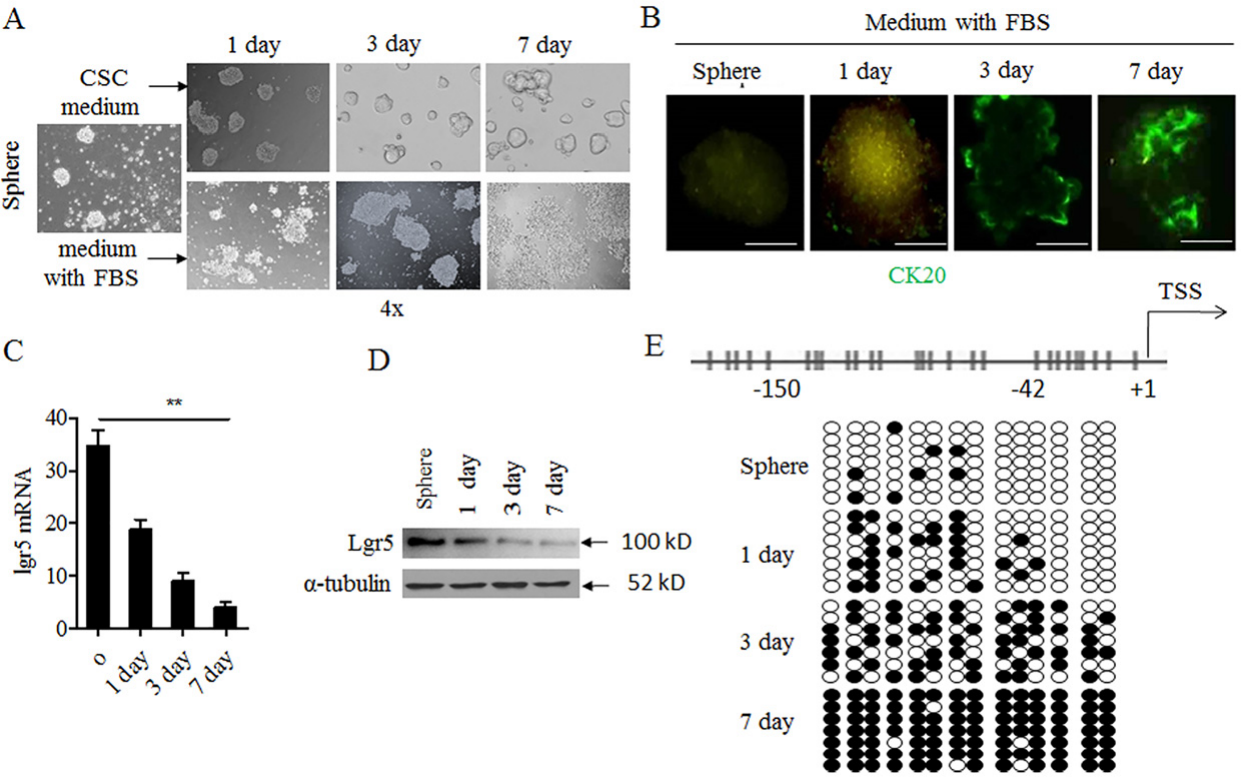
Lgr5 expression and methylation varied from CSC spheres to adherent cells. **A**. CSC spheres from HCT116 cell lines grew with CSC medium without serum (upper panel); Or, CSC spheres changed to normal medium with 10% FBS (down panel). Observation time point: 1 day, 3 day. And 7 day and photographed under light microscope. **B**. Immunofluorescent staining of CK20 in CSC spheres; Green indicated ck20 staining. **C**. RNA was collected in different time point and was analyzed by qPCR in medium with 10% FBS group. **D**. The protein was collected in different time point in medium with 10% FBS group, lgr5 was detected with western blot.westernblot α-tubulin was used as internal control. **E**. BSP analysis of lgr5 promoter methylation density in CSC spheres and time point in in medium with 10% FBS group. Black circle, methylated CpG site; white circle, unmethylated CpG site.

As reported previously, lgr5 expression was higher in undifferentiated cells as compared to differentiated cells in colorectal cancer[9]. To investigate the involvement of lgr5 DNA methylation in CSC differentiation, we derived cancer stem or stem-like cells from HCT116 cell line following a spheres model as described[41] where breast cancer stem cells were enriched by culturing in suspension as nonadherent spheres. Two weeks after culture in serum-free medium containing EGF and FGF-2, we obtained CSC spheres from HCT116 cell line (Fig 2A, first panel). No expression of cytokeratin 20 (CK20), a marker for epithelial cell differentiation, was detectable in the CSC spheres by IF (Fig 2B, first panel). Then, these CSC spheres could grow by suspension condition, and gradually became tight and merged together (Fig 2, upper panel). But these spheres were incubated with serum-containing medium where they gradually adhered to the surface of culture plate, which was the characteristic of differenation cellsby dissociation of cells from the spheres (Fig 2A, down panel), and up-regulation of CK20 (Fig 2B) from day 0 to day 7. Concomitant with the differentiation process, lgr5 expression was also reduced by qPCR and western blot (Fig 2C and 2D). The down-regulation of lgr5 expression also well correlated with a significant increase in the density of promoter methylation (Fig 2E).

### Knocking down lgr5 induced differentiation of CSC spheres

**Fig 3:**
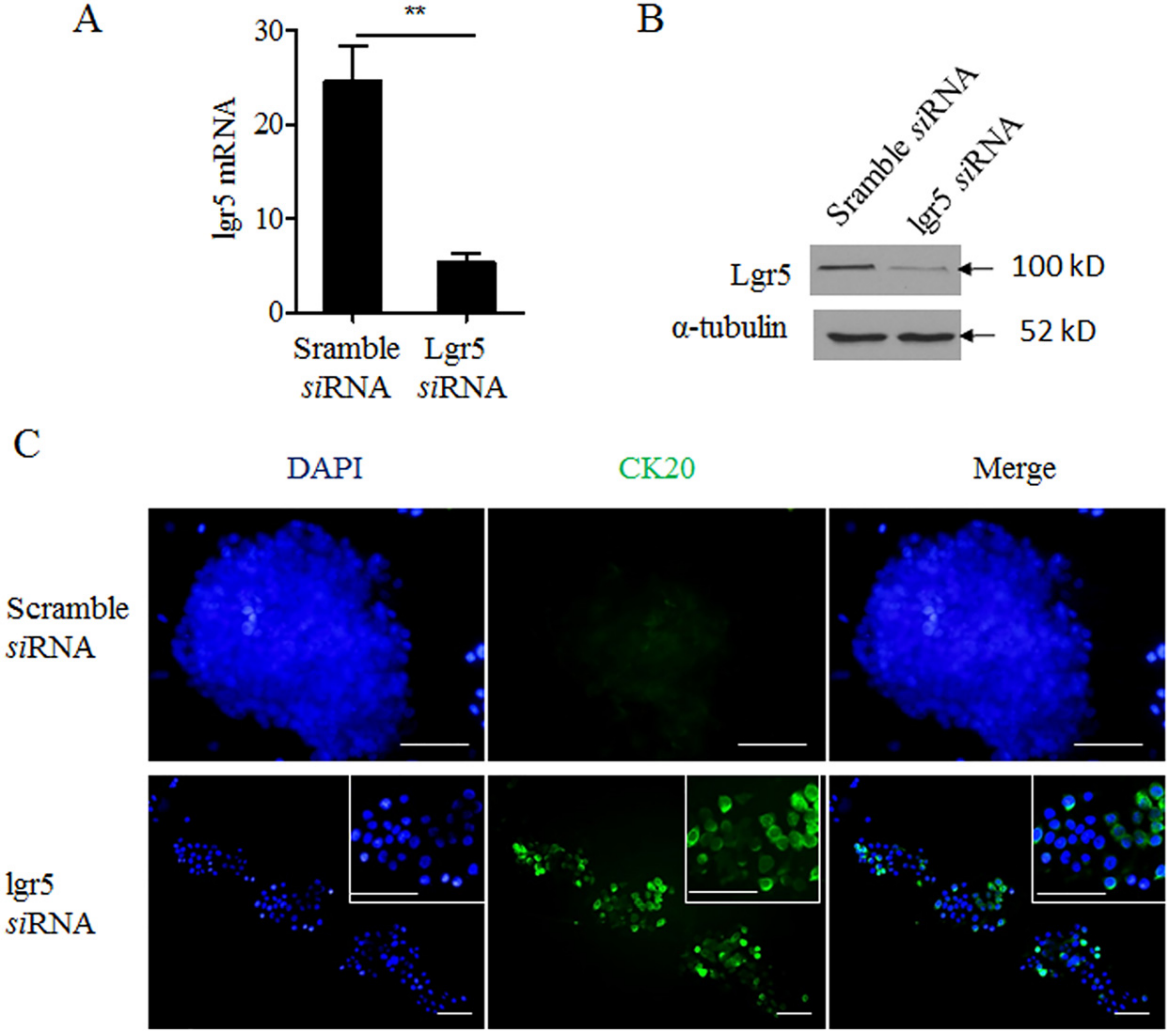
Knocking down lgr5 induced differentiation of CSC spheres. **A**. **B**.Expression of lgr5 mRNA and protein in CSC spheres transfected with either scramble or lgr5-specific siRNAwas examined by qPCR and western blot (upper panel) **C**. Immunofluorescent staining of CK20 (green) in CSC spheres transfected as in **A**. Blue: DAPI nuclear stain.

To examine the causal relationship between decreased lgr5 expression and differentiation of CSC spheres, we transfected CSC spheres with lgr5-specific siRNA. As compared to scrambled control siRNA, lgr5-specific siRNA significantly reduced the lgr5 expression (Fig 3A, p < 0.001). The siRNA treatment also led to up-regulation of CK20, as well as dissociation of CSC spheres (Fig 3B). This is consistent with the phenotypes associated with CSC differentiation, suggesting that down-regulation of lgr5 was sufficient to induce the differentiation of CSC spheres.

### Lgr5 methylation was detectable in colorectal cancers but not normal colon tissues

**Fig 4:**
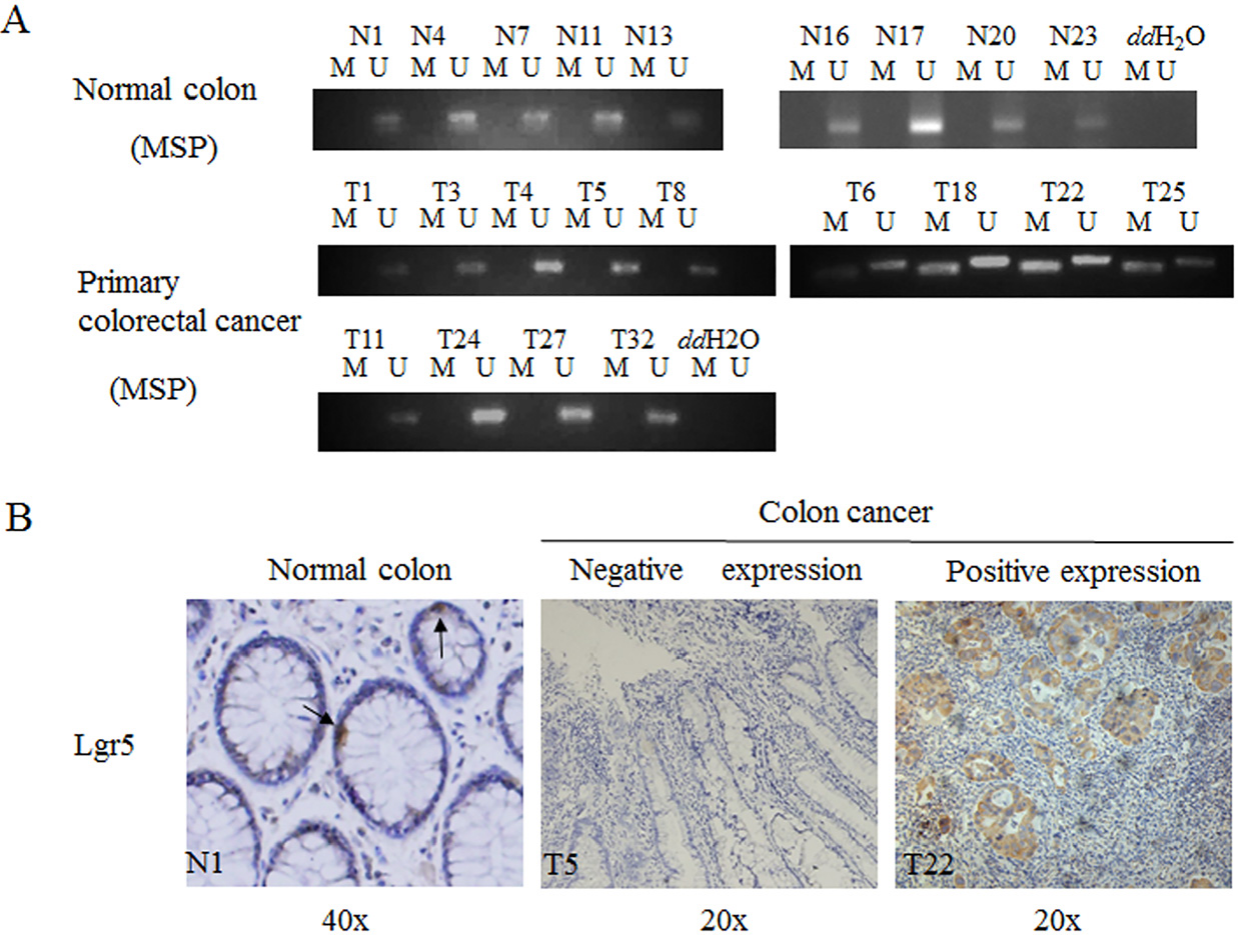
Lgr5 methylation was in normal colon tissues and primary colorectal cancers. **A**. MSP was performed on normal colon tissues (A) or primary colorectal cancers. M, methylation signal; U, unmethylation signal. **B**. Lgr5 expression was in normal tissue and cancer tissues, dark arrows represented the lgr5 positive cell in by Immunohistochemical staining. N1 represented the one of the normal colon tissues; T5 and T22 respectively represented the colon cancer patients.

Next, we investigated promoter methylation of lgr5 gene in normal colon tissues versus colorectal cancer tissues. As shown in Fig 4A and 4B, positive methylation was only detectable in colorectal cancer tissues but not normal colon tissues. MSP analysis on total 169 colorectal cancers revealed that 67 samples (40%) were positive for lgr5 promoter methylation. In addition, we also examined the lgr5 expression by IHC.Lgr5 negative expression was associated with lgr5 methylation in the same cancer patients tissue by MSP. And low methylation or unmethlation lgr5 expression was obviously higher in low methylation or unmethlation group by MSP (Fig 4C). There data suggested that lgr5 methylation was closed to its expression.

### Lgr5 methylation negatively correlated with tumor grade, tumor metastasis and positively with good prognosis of colorectal cancer

**Fig 5:**
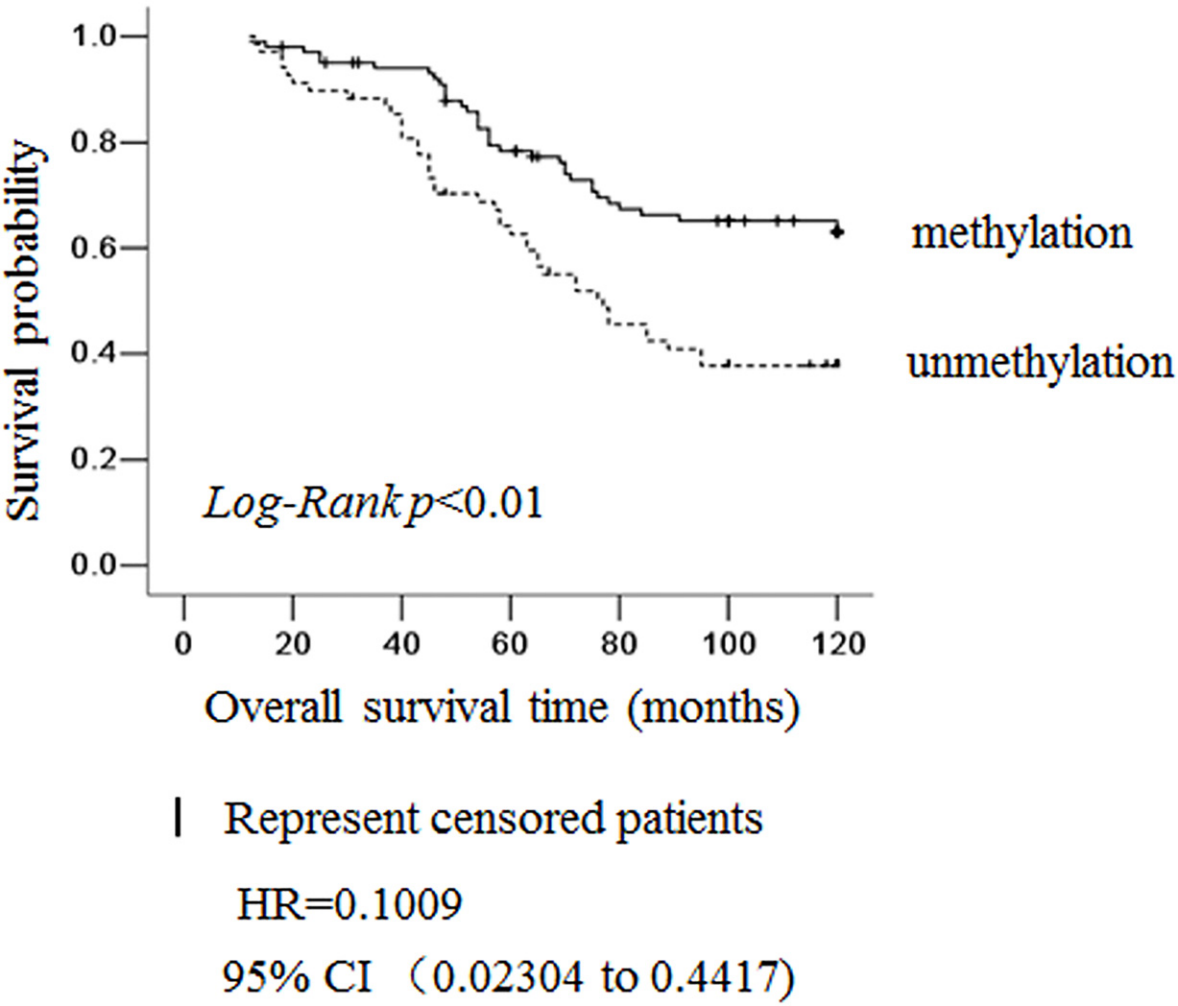
Lgr5 methylation status was an independent prognosis marker for colorectal cancer. Kaplan-Meier survival curves for patients grouped based on lgr5 methylation status. Solid line: colorectal cancer positive for lgr5 methylation, n = 67; dotted line:colorectal cancer negative for lgr5 methylation, n = 102. HR: Hazard Ratio; 95% CI: 95% Confidence interval.

To evaluate the clinical significance of lgr5 methylation, we analyzed the association between lgr5 promoter methylation status, as determined by MSP, with a variety of clinicopathological features of patients with colorectal cancer (Table 2). We identified a significant reverse correlation between positive lgr5 methylation and higher tumor grade (p<0.001), positive nodal involvement (p = 0.04) and positive distant metastasis (p = 0.024), However, we did identify not other features such as patient age, gender, tumor position or Dukes staging. The reverse correlations suggested that lgr5 methylation was related to cancer of a less invasive phenotype, *i*.*e*., higher tumor grade, negative lymph node and distant metastasis, which was further supported by the Kaplan-Meier survival analysis (Fig 5). Among the 169 patients analyzed, the 100-month survival rate was significantly higher in cases with lgr5 methylation than those unmethylation (*p =* 0.001). These results indicated that lgr5 methylation was an independent predictive biomarker for good prognosis of colorectal cancer.

**Table 2:** Correlation of lgr5 methylation with clinicopathological features of colorectal cancer patients.

Clinicopathological feature	n	Lgr5 methylation (%)	χ^2^	p
**Total**	169	67 (40%)		
**Gender**				
Female	68	30 (44%)	0.951	0.341
Male	101	37 (37%)		
**Age (years)**				
≥ 60	75	27 (36%)	0.749	0.431
< 60	94	40 (43%)		
**Tumor position**				
Rectum	109	45 (41%)	4.899	0.179
Left colon	19	4 (21%)		
Transverse colon	19	6 (32%)		
Right colon	23	12 (52%)		
**Dukes stage**				
A	51	21 (33%)	5.618	0.132
B	56	27 (36%)		
C	31	12 (39%)		
D	31	7 (25%)		
**Tumor grade**				
High	85	47 (31%)	18.84	<0.001
Moderate	52	15 (38%)		
Low	32	5 (16%)		
**Nodal status**				
Positive	31	12 (17.9%)	0.014	0.04
Negative	138	55(39%)		
**Distal metastasis**				
Positive	31	7 (25%)	4.620	0.024
Negative	138	60(51.1%)		

**Note:** p value was calculated using Pearson’s χ^2^ test.

## Discussion

Tissue self-renewal is maintained by stem cell niches. The methylation pattern within the niches regulates stem cell turnover, affecting cell fate, heterogeneity, and so on. When the balance of this pattern is perturbed, stem cells with phenotypic and genetic alterations may undergo tumorigenesis. A great amount of evidence has demonstrated that epigenetic changes, such as hypermethylation of CpG islands within promoter regions of various genes, are likely major contributors to the tumorigenesis in a wide range of cancers[42] and CpG island shores in sequences up to 2 kb distant was strongly associated with gene expression in colon cancer[43]. In addition, several cancer stem cell surface markers were also regulated by gene methylation. Furhtermore, Hypermethylation status was related to low or no expression and cell differentiation, for example, CD133 in colorectal cancer and glioma[44, 45], and EPcam in breast cancer[36]. In the current study, we hypothesized that silenced or reduced lgr5 expression was related to CpG island methylation. In support of this hypothesis, treatment with 5-aza-2’-deoxycytidine restored lgr5 expression in a couple of colorectal tumor cell lines, with dramatic up-regulation in HCT116 cells that contain completely methylated lgr5, and middle or no upregulation in other cell lines containing partially or hardly methylated lgr5. Moreover, treatment of DAC did not alter lgr5 expression in Melo published study[34] in SW620 cell line, but a little increase in our current study. We analyzed lgr5 methylation in about ~1kb promoter of lgr5, and it might be some CpG islands in other >1kb fragment. So we can`t detect the lgr5 methylation in SW620 cell line, but lgr5 expression was a little increase after DAC treatment or other unclear cause. These data suggested that DNA methylation played an important role in regulating lgr5 expression.

Previous studies showed that lgr5 played a key role in maintaining the properties of intestinal and colon stem cells. Low or no lgr5 expression was detected in differentiated cells. In contrast, a high expression of CK20, a differentiated cell marker, was also found in different cancers [38, 46]. Consistent with these findings, our results indicated that lgr5 expression was high in colorectal CSC, and reduced differentiation, which was accompanied by enhanced DNA methylation and CK20 up-regulation. Furthermore, knocking down lgr5 in CSC spheres induced CSC differentiation. These results suggested that down-regulation of lgr5, which resulted from DNA methylation, was responsible for the differentiated phenotype of CSC.

We further investigated the clinical significance of lgr5 methylation in sporadic colorectal cancer. We noticed that this was not the first study looking into epigenetic modification of CSC markers. It has been reported that hypermethylation of the CpG islands located in the promoter region of the stem/precursor cell marker CD44, one of the colorectal and other cancer stem cell surface markers, might predict biochemical recurrence in prostate cancer patients undergoing radical prostatectomy[47]. The hypomethylation of CpG sites in three proximal promoters determined CD133 expression in glioblastomas[42, 48] (delet: and hypermethylation of CD133 in HCT116 led to xenograft tumor formation in nude mice as compared to cells without CD133 methylation). Interestingly, we also observed that lgr5 was more frequently methylated in colorectal cancer colons than in normal colons, and that lgr5 expression was tightly regulated by methylation. Clinicopathological analysis unraveled the reverse association of positive lgr5 methylation with higher tumor grade and invasiveness, in agreement with the notion that over-expression of lgr5 was associated with the more malignant and invasive cancers. In addition, we also found that lgr5 methylation was significantly associated with better prognosis among colorectal cancer patients.

It was previously reported that loss of lgr5 expression is seen in approximately 40% colorectal cancer [22]. In our study, we also observed positive lgr5 promoter methylation in approximately 40% of colorectal cancers, and verified that negative lgr5 expression was significantly associated with the promoter DNA methylation. These results imply that lgr5 provides a balance between cancer cell proliferation and CSC differentiation. Therefore, deregulated lgr5 expression may tip the balance toward a more transformed and oncogenic phenotype, as observed in another stem-cell marker, Oct4 [49].Similarly, lgr5 deficiency in normal small intestine leads to premature Paneth cells differentiation in the small intestine [27]. In this study, we identified a novel mechanism to control the expression of lgr5, *i*.*e*. via promoter methylation. We demonstrated that the down-regulation of lgr5, as a result of either siRNA treatment or promoter methylation, was sufficient to induce CSC differentiation. Similar regulations have been observed in other stem cell markers, such as Oct4 [49]. It has been suggested that effective DNA methylation is in place to ensure silencing of this potent cancer gene during development. Hypermethylation of lgr5 results in cancer stem cell differentiation. As we know, differentiated cells aren’t capable of potent self-renewal and aresensitive to traditional chemotherapy drugs, so lgr5 methylation might enhance the chemotherapy that is sensitive to the colorectal cancer cell. However, it’s noteworthy that DNA methylation is not the sole molecular mechanism regulating cancer stem cell heterogeneity. Other mechanisms, such as histone modifications, microRNA, chromatin remodelers and so on[50], may also function during carcinogenesis, which are beyond the scope of this study. What’s more is that DNA methylation may have a crosstalk with other signaling pathways or regulation of other gene expression. Melo [34] *et al*. reported that promoter methylation of Wnt target genes is a strong predictor for recurrence of colorectal cancer, and that a highly possible mechanism is that inhibition of these genes resulted from methylation might factually enhance Wnt activity levels and lead to disease progression and relapse via activation of yet undiscovered positive targets. The other hand, Lgr5 high expression was related to the poor prognosis in colon cancer [51, 52]. Silencing lgr5 expression could promote colon cancer cell apoptosis and reduced the metastasis [22]. The methylation of the promoter reduced the lgr5 expression in human colon cancer tissue and cell lines in Melo et al. report and in our study. It seemed that lgr5 methylation indicated the good prognosis and less invasion, and our results was similar to this. There was somewhat different with Melo et al. results, it may be the bias of our samples. The metastasis samples is 31 (only 18.3%, 31/169) in our study, but the samples almost were stage II in Melo`s article.

Overall, our study showed that lgr5 methylation isresponsible for lgr5 gene silencing and cancer stem cell differentiation in colorectal cancer. Moreover, lgr5 methylation is also reversely associated with high tumor grade and positive distant metastasis. Moreover, it may serve as a marker to predict better survival among patients with primary colorectal cancer. These data suggest that the silencingof lgr5 gene via CpG island methylation may be involved in the progression of CRC and may potentially serve as a therapeutic target (a supplementary to traditional chemotherapy for colorectal cancer). Further studies are necessary to elucidate the detailed regulatory mechanisms in vivo.

## Supporting Information

S1 Data Data for survival analysis (extended Fig 5). (XLSX)
